# Association of Birth Defects With Child Mortality Before Age 14 Years

**DOI:** 10.1001/jamanetworkopen.2022.6739

**Published:** 2022-04-11

**Authors:** Marie-Laure Sattolo, Laura Arbour, Marianne Bilodeau-Bertrand, Ga Eun Lee, Chantal Nelson, Nathalie Auger

**Affiliations:** 1 Department of Epidemiology, Biostatistics, and Occupational Health, McGill University, Montreal, Quebec, Canada; 2 Institut national de santé publique du Québec, Montreal, Quebec, Canada; 3 Department of Medical Genetics, University of British Columbia, Vancouver, British Columbia, Canada; 4 University of Montreal Hospital Research Centre, Montreal, Quebec, Canada; 5 Maternal and Infant Health Surveillance Section, Public Health Agency of Canada, Ottawa, Ontario, Canada; 6 Department of Social and Preventive Medicine, School of Public Health, University of Montreal, Montreal, Quebec, Canada

## Abstract

**Importance:**

Causes of death in children with birth defects are poorly understood.

**Objective:**

To determine mortality rates by cause of death in children with and without birth defects.

**Design, Setting, and Participants:**

This longitudinal cohort study included a population-based sample of 1 037 688 children and was conducted in all hospitals in Quebec, Canada, with 7 700 596 person-years of follow-up between birth and age 14 years (April 1, 2006, to March 31, 2020).

**Exposures:**

Presence or absence of a birth defect.

**Main Outcomes and Measures:**

Outcomes were all-cause and cause-specific mortality. Hazard ratios (HRs) and 95% CIs were computed for the association between birth defects and mortality with Cox proportional hazards models adjusted for patient characteristics.

**Results:**

Among the 1 037 688 children in the cohort, 95 566 had birth defects (56.5% boys). There were 532 542 boys in the cohort (51.3%), and mean (SD) age at the end of follow-up was 7.42 (3.72) years. There were 918 deaths among children with defects, and the mean (SD) age was 0.93 (2.07) years at death; there were 1082 deaths among the 942 122 children without defects, and the mean (SD) age at death was 0.50 (1.51) years. Mortality rates were higher for children with birth defects compared with no defect (1.3 vs 0.2 deaths per 1000 person-years, respectively). Girls (HR, 5.66; 95% CI, 4.96-6.47) and boys (HR, 4.69; 95% CI, 4.15-5.29) with birth defects had an elevated risk of death before 14 years compared with unaffected children. Birth defects were associated with mortality from circulatory (HR, 26.59; 95% CI, 17.73-39.87), respiratory (HR, 23.03; 95% CI, 15.09-35.14), and digestive causes (HR, 31.77; 95% CI, 11.87-85.04), but anomalies were rarely listed as the cause of death. Compared with children with no defect, those with birth defects were at greatest risk of death between 28 and 364 days of life.

**Conclusions and Relevance:**

This cohort study of 1 037 688 children suggests that birth defects were strongly associated with mortality owing to circulatory, respiratory, and digestive causes. This finding suggests that the contribution of birth defects may be underestimated in mortality statistics.

## Key Points

**Question:**

To what extent are birth defects associated with child mortality?

**Findings:**

In this cohort study of 1 037 688 children younger than 14 years, birth defects were associated with more than 23 times the risk of mortality from circulatory, respiratory, and digestive causes compared with no defect. Risk of death was elevated for children with central nervous system, heart, and chromosomal defects, and between 28 and 364 days of life.

**Meaning:**

These findings suggest that children with birth defects have a high risk of postneonatal mortality, including mortality from cardiorespiratory and digestive causes.

## Teaser

This cohort study uses the Maintenance and Exploitation of Data for the Study of Hospital Clientele data set to examine mortality rates by cause of death in children with and without birth defects.

## Introduction

Children with birth defects have high mortality,^1^ but cause of death is not always clear. Birth defects account for 20% of infant deaths in the United States.^2^ Preventing mortality is challenging because causes of death in children with birth defects are often poorly understood. Although it is easy to identify defects that are incompatible with life, severe anomalies are rare and account for only a fraction of deaths.^1,3,4^ For the majority of children with birth defects, mortality may not be directly due to the anomaly.^5,6^ Improved understanding of cause of death is needed to improve the survival of children with birth defects.

Most studies of child mortality rely on death certificates or cross-sectional data with incomplete information on birth defects^2,7,8,9^ and may underestimate mortality due to congenital anomalies.^6^ Cohorts of children with birth defects frequently lack a comparison group.^1,3,10^ In a systematic review of 55 studies of long-term survival, only 7 studies of children with birth defects had a comparison group.^1^ The review confirmed that children with birth defects had an elevated risk of mortality, but causes of death were not investigated.^1^ The only study that assessed specific causes of mortality attributed deaths primarily to perinatal conditions and the underlying anomaly.^5^ However, the study assessed births from the 1970s and 1980s.^5^ It is likely that the findings are outdated because recent advances in the management of birth defects may have improved survival since then. To provide updated data, we studied the association between birth defects and cause-specific mortality in a longitudinal cohort of children with 14 years of follow-up between April 1, 2006, and March 31, 2020.

## Methods

### Study Design and Population

In this cohort study, we analyzed 1 037 688 children who were born in hospitals in Quebec, Canada, between 2006 and 2019. Approximately 98% of births in Quebec occur in a hospital; thus, the cohort was representative of the population. We followed the children from birth to age 14 years, using health insurance numbers to identify in-hospital mortality. Data on race and ethnicity were not available for this study. We observed the Strengthening the Reporting of Observational Studies in Epidemiology (STROBE) reporting guideline for cohort studies (eTable 1 in the Supplement).

We derived data from the Maintenance and Use of Data for the Study of Hospital Clientele data set, which contains discharge summaries for all hospitalizations in Quebec.^11^ The data include diagnostic and procedure codes for birth defects and the cause of death, as well as maternal and infant demographic information.^12^ We did not include stillbirths and pregnancy terminations. The institutional review board of the University of Montreal Hospital Centre waived informed consent and ethics review because the data were deidentified.

### Birth Defects

The main exposure measure was the presence of a birth defect, defined as a structural and functional anomaly with or without an underlying genetic anomaly. A large number of birth defects in Quebec are detected by universal ultrasonographic screening between 18 and 22 weeks of pregnancy.^12^ Birth defects may also be discovered during first-trimester dating ultrasonography, third-trimester growth ultrasonography, and clinical or radiographic examination at birth and during childhood. We coded birth defects with the *International Statistical Classification of Diseases and Related Health Problems, 10th Revision* (*ICD-10*) (eTable 2 in the Supplement). We increased the ascertainment of cases by identifying children who received corrective procedures for defects following surgical codes in the Canadian Classification of Health Interventions.

We categorized defects by organ system: heart; central nervous system; orofacial cleft; eye, ear, and nose; respiratory; digestive; abdominal wall; urinary; genital; musculoskeletal; chromosomal; and other. We examined different defects, such as hypoplastic left heart syndrome, neural tube defects, Down syndrome, and clubfoot. We further identified children with multiple or isolated birth defects. We considered children who had defects in more than 1 organ system as having multiple birth defects. The comparison group included children without birth defects.

### Cause of Death

The main outcome was in-hospital mortality before age 14 years. In Quebec, child deaths typically occur in the hospital or during transport to the hospital while resuscitation is attempted. The data capture nearly all child deaths in the province. Although underlying causes were not available, the immediate cause of death is recorded on the discharge summary with *ICD-10* codes. The immediate cause of death is valuable to identify interventions that may prolong survival.

We categorized deaths by cause, following chapters in *ICD-10:* infection (A00-B99); cancer (C00-D48); blood (D50-D89); endocrine, nutritional, and metabolic (E00-E90); nervous system (G00-G99); circulatory (I00-I99); respiratory (J00-J99); digestive (K00-K93); musculoskeletal (M00-M99); genitourinary (N00-N99); and perinatal (P00-P96). We included causes relating to congenital anomalies (Q00-Q99), shock and ill-defined conditions (R00-R99), injury and external causes (S00-T98 and V01-Y98), and health status and contact with health services (Z00-Z99).

### Covariates

We adjusted for potential confounders associated with child mortality in the literature,^5,13^ including maternal age (<25, 25-34, and ≥35 years), parity (0, 1, and ≥2 previous deliveries), multiple birth, preterm birth (<37 and ≥37 gestational weeks), sex, socioeconomic deprivation (most disadvantaged quintile of neighborhoods according to levels of employment, income, and education), and period at birth (2006-2009, 2010-2014, and 2015-2019). Data on socioeconomic deprivation were missing for 4.0% of children; we placed this variable in a separate category because there was no difference in the distribution between exposed and unexposed children.

### Statistical Analysis

We calculated mortality rates and used Cox proportional hazards models to compute hazard ratios (HRs) and 95% CIs for the association between birth defects and mortality in boys and girls separately. We ran models for each type of defect and examined both all-cause and cause-specific mortality. The time scale was measured as the number of days between birth and the date of death or study end on March 31, 2020. We censored children who survived to the end of the study. We adjusted the models for maternal age, parity, multiple birth, preterm birth, socioeconomic deprivation, and period. We accounted for children with the same mother by using robust error estimators and examined the proportionality of hazards with log (−log survival) curves.

In secondary analyses, we stratified the analysis by age at death (<1 day, 1-27 days, 28-364 days, 1-4 years, and 5-14 years). In sensitivity analyses, we restricted the data to term births to rule out any association with prematurity. We performed the analysis in SAS version 9.4 (SAS Institute Inc) and assessed statistical significance with 95% CIs.

## Results

Among 1 037 688 children in the cohort (532 542 boys [51.3%] and 505 146 girls [48.7%]), 95 566 had at least 1 birth defect (9%), and there were 2000 deaths (0.2%) during 7 700 596 person-years of follow-up (Table 1). Mean (SD) age at the end of follow-up was 7.42 (3.72) years. There were 918 deaths among children with defects, for a mortality rate of 1.3 (95% CI, 1.2-1.5) per 1000 person-years for girls and 1.2 (95% CI, 1.1-1.3) per 1000 person-years for boys. Among the 942 122 children without defects, there were 1082 deaths, for a mortality rate of 0.2 (95% CI, 0.2-0.2) per 1000 person-years for boys and 0.1 (95% CI, 0.1-0.2) per 1000 person-years for girls. Overall, 46% of children who died before age 14 years had a birth defect. Among children who died, mean (SD) age at death was 0.93 (2.07) years for those with birth defects and 0.50 (1.51) years for those without them. Children with and without defects had similar distributions of maternal age, parity, and socioeconomic deprivation (eTable 3 in the Supplement). There were more preterm births among children with defects compared with those with no defect (13.1% vs 6.1%). Boys tended to have more defects than girls (56.5% vs 43.5%).

**Table 1.** Association Between Birth Defects and Mortality Before Age 14 Years

Type of defect^a^	No. of deaths/No. of children with defect (%)		Mortality rate per 1000 person-years (95% CI)		Hazard ratio (95% CI)^b^	
	Boys	Girls	Boys	Girls	Boys	Girls
Any	495/53 954 (0.9)	423/41 612 (1.0)	1.2 (1.1-1.3)	1.3 (1.2-1.5)	4.69 (4.15-5.29)	5.66 (4.96-6.47)
Heart	306/10 438 (2.9)	288/9576 (3.0)	4.2 (3.8-4.7)	4.3 (3.9-4.9)	8.36 (7.23-9.67)	9.18 (7.85-10.72)
Central nervous system	113/2550 (4.4)	101/2312 (4.4)	6.3 (5.2-7.5)	6.3 (5.2-7.6)	10.57 (8.60-12.99)	12.09 (9.70-15.06)
Orofacial cleft	21/1045 (2.0)	16/861 (1.9)	2.7 (1.8-4.2)	2.5 (1.5-4.0)	10.73 (6.93-16.59)	12.35 (7.50-20.34)
Eye, ear, nose	39/8769 (0.4)	29/7637 (0.4)	0.5 (0.4-0.7)	0.5 (0.3-0.7)	2.91 (2.11-4.03)	2.85 (1.96-4.14)
Respiratory	63/2613 (2.4)	48/1845 (2.6)	3.6 (2.8-4.6)	4.0 (3.0-5.3)	7.46 (5.74-9.69)	9.44 (7.00-12.73)
Digestive	81/3948 (2.1)	61/2725 (2.2)	2.8 (2.2-3.4)	3.1 (2.4-3.9)	7.86 (6.22-9.93)	8.22 (6.29-10.75)
Abdominal wall^c^	36/723 (5.0)	28/682 (4.1)	7.0 (5.1-9.8)	5.8 (4.0-8.4)	11.50 (8.17-16.18)	10.92 (7.41-16.09)
Urinary	77/9097 (0.8)	52/8099 (1.1)	1.2 (1.0-1.5)	1.6 (1.2-2.1)	4.16 (3.28-5.28)	6.08 (4.56-8.11)
Genital	36/8099 (0.4)	<5	0.6 (0.4-0.8)	0.5 (0.2-1.4)	2.23 (1.59-3.13)	2.02 (0.76-5.42)
Musculoskeletal	83/12 451 (0.7)	69/13 758 (0.5)	0.9 (0.7-1.1)	0.7 (0.5-0.8)	4.11 (3.26-5.17)	4.00 (3.10-5.14)
Chromosomal	72/1228 (5.9)	66/1108 (6.0)	8.6 (6.8-10.8)	8.5 (6.7-10.8)	17.75 (13.84-22.77)	21.94 (16.85-28.57)
Other	79/3055 (2.6)	76/2676 (2.8)	3.4 (2.7-4.2)	3.8 (3.1-4.8)	11.46 (9.05-14.52)	14.23 (11.15-18.16)
Multiple	225/6240 (3.6)	187/4255 (4.4)	5.1 (4.4-5.8)	6.3 (5.4-7.2)	10.14 (8.64-11.90)	13.19 (11.06-15.73)
Isolated	270/47 714 (0.6)	236/37 357 (0.6)	0.8 (0.7-0.8)	0.8 (0.7-0.9)	3.06 (2.65-3.54)	3.67 (3.13-4.29)
None	589/478 588 (0.1)	493/463 534 (0.1)	0.2 (0.2-0.2)	0.1 (0.1-0.2)	1 [Reference]	1 [Reference]

^a^ Categories are not mutually exclusive.

^b^ Hazard ratio for birth defect vs no defect, adjusted for maternal age, parity, multiple birth, preterm birth, socioeconomic deprivation, and period.

^c^ Gastroschisis, omphalocele, diaphragmatic hernia, and other.

Children with birth defects had a high risk of mortality between birth and age 14 years (Table 1; eTable 4 in the Supplement). Compared with having no defect, boys with birth defects had 4.69 times the risk of death (95% CI, 4.15-5.29), whereas girls had 5.66 times the risk (95% CI, 4.96-6.47). The highest risks of mortality were among children with chromosomal anomalies (HR for boys, 17.75 [95% CI, 13.84-22.77]; HR for girls, 21.94 [95% CI, 16.85-28.57]). Children with multiple birth defects had a higher risk of death than those with isolated defects (HR for boys, respectively, 10.14 [95% CI, 8.64-11.90] vs 3.06 [95% CI, 2.65-3.54]; HR for girls, respectively, 13.19 [95% CI, 11.06-15.73] vs 3.67 [95% CI, 3.13-4.29]).

Several specific defects had particularly strong associations with mortality (Table 2). Hypoplastic left heart syndrome was associated with more than 146 times the risk of mortality before age 14 years (HR for boys, 145.89 [95% CI, 105.25-202.22]; HR for girls, 171.80 [95% CI, 117.87-250.41]), transposition the of great vessels with more than 48 times the risk (HR for boys, 48.43 [95% CI, 31.30-74.94]; HR for girls, 59.62 [95% CI, 36.71-96.83]), diaphragmatic hernia with more than 36 times the risk (HR for boys, 38.59 [95% CI, 24.09-61.82]; HR for girls, 35.87 [95% CI, 20.16-63.82]), and trisomy 13 and 18 with more than 69 times the risk (HR for boys, 69.53 [95% CI, 39.70-121.77]; HR for girls, 122.61 [95% CI, 81.27-184.99]). Risks were generally similar for boys and girls.

**Table 2.** Association Between Specific Types of Birth Defects and Mortality Before Age 14 Years

Type of defect^a^	Mortality rate per 1000 person-years (95% CI)		Hazard ratio (95% CI)^b^	
	Boys	Girls	Boys	Girls
Critical heart	11.9 (9.6-14.6)	15.3 (12.3-19.1)	42.02 (33.60-52.55)	48.00 (37.67-61.17)
Transposition of the great vessels	9.7 (6.3-14.8)	14.5 (9.0-23.4)	48.43 (31.30-74.94)	59.62 (36.71-96.83)
Tetralogy of Fallot	5.1 (2.8-9.1)	13.0 (8.6-19.6)	14.17 (7.79-25.77)	35.48 (23.30-54.04)
Hypoplastic left heart syndrome	63.8 (46.8-86.9)	102.3 (71.5-146.3)	145.89 (105.25-202.22)	171.80 (117.87-250.41)
Coarctation of aorta	7.1 (4.5-11.2)	5.6 (2.9-10.8)	28.10 (17.78-44.42)	16.96 (8.75-32.88)
Other critical^c^	20.9 (14.4-30.2)	20.1 (13.2-30.5)	67.16 (45.81-98.48)	54.07 (35.05-83.41)
Noncritical heart	4.1 (3.6-4.6)	4.2 (3.7-4.7)	7.94 (6.84-9.21)	8.67 (7.41-10.16)
Ventricular septum	3.5 (2.7-4.5)	3.4 (2.6-4.3)	9.76 (7.44-12.80)	9.35 (7.14-12.24)
Atrial septum	4.1 (3.5-4.9)	3.9 (3.3-4.8)	6.60 (5.40-8.06)	6.14 (4.95-7.61)
Other noncritical^d^	5.2 (4.6-5.9)	5.9 (5.2-6.7)	9.20 (7.86-10.78)	10.96 (9.28-12.93)
Neural tube^e^	3.3 (1.6-6.6)	4.4 (2.4-8.3)	10.68 (5.31-21.50)	18.99 (10.13-35.58)
Microcephaly	9.8 (7.0-13.7)	7.8 (5.6-10.7)	16.97 (11.98-24.05)	15.02 (10.71-21.07)
Hydrocephalus	14.7 (10.7-20.1)	13.2 (9.1-19.2)	21.56 (15.55-29.91)	19.37 (13.09-28.67)
Other central nervous system^f^	4.5 (3.4-5.9)	6.0 (4.6-7.9)	6.60 (4.94-8.81)	9.97 (7.49-13.26)
Eye	1.0 (0.7-1.5)	0.9 (0.6-1.4)	3.82 (2.54-5.75)	3.50 (2.19-5.61)
Ear	0.3 (0.2-0.6)	0.2 (0.1-0.4)	2.36 (1.44-3.88)	1.68 (0.83-3.38)
Nose	2.7 (1.3-5.6)	3.8 (2.1-6.9)	8.59 (4.08-18.11)	18.78 (10.32-34.18)
Lung malformation	12.5 (9.0-17.3)	13.9 (9.6-20.2)	18.24 (12.97-25.65)	21.29 (14.48-31.28)
Other respiratory^g^	2.0 (1.4-2.9)	2.1 (1.4-3.2)	4.24 (2.94-6.13)	5.37 (3.49-8.24)
Biliary or intestinal atresia	7.9 (5.7-11.2)	7.4 (5.0-11.0)	11.71 (8.22-16.67)	10.40 (6.93-15.59)
Other digestive^h^	2.3 (1.8-3.0)	2.5 (1.8-3.3)	7.17 (5.50-9.35)	7.39 (5.42-10.08)
Diaphragmatic hernia	16.3 (10.2-25.8)	14.3 (8.1-25.2)	38.59 (24.09-61.82)	35.87 (20.16-63.82)
Omphalocele	3.6 (1.4-9.6)	6.1 (3.2-11.7)	8.37 (3.12-22.40)	14.90 (7.69-28.86)
Gastroschisis	4.7 (2.3-9.3)	5.6 (2.9-10.7)	4.11 (2.04-8.30)	5.81 (2.98-11.34)
Other abdominal wall	6.4 (3.4-11.9)	2.4 (0.8-7.4)	18.01 (9.62-33.72)	9.25 (2.97-28.76)
Renal agenesis	3.4 (2.0-5.8)	4.1 (2.1-7.9)	10.64 (6.26-18.09)	13.67 (7.06-26.46)
Other urinary^i^	1.1 (0.9-1.4)	1.5 (1.1-2.0)	3.82 (2.97-4.91)	5.73 (4.24-7.74)
Female genital	NA	0.1 (0.0-1.0)	NA	0.52 (0.07-3.71)
Male genital	0.5 (0.4-0.8)	NA	2.05 (1.44-2.92)	NA
Indeterminate sex	17.1 (7.1-41.0)	9.5 (3.1-29.4)	18.28 (7.55-44.28)	15.90 (5.09-49.66)
Congenital hip dislocation	0.6 (0.3-1.3)	0.3 (0.1-0.6)	3.08 (1.46-6.49)	2.27 (1.13-4.57)
Clubfoot	0.7 (0.5-1.0)	0.3 (0.2-0.6)	3.77 (2.56-5.55)	2.46 (1.49-4.05)
Polydactyly, syndactyly	0.7 (0.3-1.3)	1.4 (0.8-2.4)	2.95 (1.53-5.70)	6.65 (3.75-11.80)
Limbs and digits	3.2 (1.5-6.7)	3.8 (1.8-8.0)	9.06 (4.29-19.10)	13.37 (6.33-28.22)
Other musculoskeletal^j^	1.9 (1.4-2.4)	1.6 (1.2-2.2)	6.24 (4.73-8.24)	6.64 (4.84-9.11)
Down syndrome	6.6 (4.3-10.0)	4.9 (2.9-8.3)	11.12 (7.22-17.14)	11.86 (6.93-20.30)
Trisomy 13 and 18	108.5 (63.0-186.8)	169.8 (115.6-249.3)	69.53 (39.70-121.77)	122.61 (81.27-184.99)
Other chromosomal	7.4 (5.3-10.2)	5.3 (3.6-7.8)	18.32 (13.11-25.60)	14.51 (9.75-21.58)
None	0.2 (0.2-0.2)	0.1 (0.1-0.2)	1 [Reference]	1 [Reference]

Abbreviation: NA, not applicable.

^a^ Categories are not mutually exclusive.

^b^ Hazard ratio for birth defect vs no defect, adjusted for maternal age, parity, multiple birth, preterm birth, socioeconomic deprivation, and period.

^c^ Common truncus, common ventricle, pulmonary valve atresia, congenital tricuspid atresia, Ebstein anomaly, and total anomalous pulmonary venous connection.

^d^ Endocardial cushion, valve, other aorta, and pulmonary artery defects; heterotaxy; patent ductus arteriosus; cor triatriatum; congenital stenosis of vena cava; persistent left superior vena cava; and other.

^e^ Anencephaly, encephalocele, and spina bifida.

^f^ Malformations of brain (congenital malformations of corpus callosum, arrhinencephaly, holoprosencephaly, and other) and spinal cord (amyelia, diastematomyelia, and other).

^g^ Malformations of larynx, trachea, bronchus, and pleura; mediastinal cyst; and other.

^h^ Malformations of lips, tongue, salivary gland, palate, mouth, pharyngeal pouch, and pharynx; Meckel diverticulum; macroglossia; tracheoesophageal fistula without atresia; esophageal stenosis and stricture; and other.

^i^ Cystic kidney, obstructive defects of renal pelvis, and other.

^j^ Deformities of hand and knee; bowing of femur, tibia, and fibula; craniosynostosis; craniofacial dysostosis; hypertelorism; Klippel-Feil syndrome; congenital scoliosis; osteogenesis imperfecta; and other.

Among the 918 children with birth defects who died, the most common causes of death were perinatal conditions (330; 35.9%) (Table 3). Perinatal conditions were frequent causes of death among children with heart (224 of 594; 37.7%), central nervous system (49 of 214; 22.9%), digestive (42 of 142; 29.6%), abdominal wall (23 of 64; 35.9%), urinary (38 of 129; 29.5%), genital (15 of 40; 37.5%), musculoskeletal (51 of 152; 33.6%), and chromosomal (39 of 138; 28.3%) defects. Anomalies were frequently the cause of death among children with orofacial cleft (11 of 37; 29.7%); eye, ear, and nose (17 of 68; 25.0%); and respiratory (38 of 111; 34.2%) defects. Among children without defects, perinatal conditions were much more commonly the cause of death (832 of 1082; 76.9%).

**Table 3.** Distribution of Cause of Death of 918 Children With and 1082 Children Without Birth Defects

Type of defect^a^	No. of deaths by cause (%)								
	Congenital anomaly	Infection	Cancer	Nervous system	Circulatory	Respiratory	Digestive	Perinatal	Other
Any	200 (21.8)	15 (1.6)	36 (3.9)	49 (5.3)	95 (10.3)	85 (9.3)	22 (2.4)	330 (35.9)	86 (9.4)
Heart	127 (21.4)	11 (1.9)	12 (2.0)	27 (4.5)	78 (13.1)	50 (8.4)	9 (1.5)	224 (37.7)	56 (9.4)
Central nervous system	48 (22.4)	<5	23 (10.7)	18 (8.4)	16 (7.5)	39 (18.2)	5 (2.3)	49 (22.9)	15 (7.0)
Orofacial cleft	11 (29.7)	0	0	<5	5 (13.5)	7 (18.9)	0	8 (21.6)	<5
Eye, ear, nose	17 (25.0)	<5	<5	7 (10.3)	5 (7.4)	12 (17.6)	<5	14 (20.6)	7 (10.3)
Respiratory	38 (34.2)	<5	<5	<5	9 (8.1)	19 (17.1)	<5	25 (22.5)	7 (6.3)
Digestive	29 (20.4)	<5	<5	5 (3.5)	11 (7.7)	17 (12.0)	12 (8.5)	42 (29.6)	21 (14.8)
Abdominal wall	23 (35.9)	<5	0	<5	<5	<5	5 (7.8)	23 (35.9)	<5
Urinary	31 (24.0)	<5	<5	10 (7.8)	8 (6.2)	20 (15.5)	<5	38 (29.5)	12 (9.3)
Genital	6 (15.0)	0	<5	<5	<5	6 (15.0)	<5	15 (37.5)	<5
Musculoskeletal	30 (19.7)	<5	<5	7 (4.6)	11 (7.2)	24 (15.8)	6 (3.9)	51 (33.6)	20 (13.2)
Chromosomal	39 (28.3)	<5	<5	6 (4.3)	23 (16.7)	14 (10.1)	<5	39 (28.3)	8 (5.8)
None	0	8 (0.7)	34 (3.1)	53 (4.9)	32 (3.0)	30 (2.8)	5 (0.5)	832 (76.9)	88 (8.1)

^a^ Categories are not mutually exclusive.

Birth defects were associated with an increased risk of death from most causes (Table 4). Compared with children with no defect, those with birth defects had elevated risks of mortality from circulatory (HR, 26.59; 95% CI, 17.73-39.87), respiratory (HR, 23.03; 95% CI, 15.09-35.14), and digestive causes (HR, 31.77; 95% CI, 11.87-85.04). Birth defects were also associated with mortality from infection (HR, 14.14; 95% CI, 5.91-33.85); endocrine, nutritional, and metabolic diseases (HR, 11.74; 95% CI, 5.02-27.46); and cancer (HR, 9.75; 95% CI, 6.07-15.64). Birth defects were not associated with mortality from genitourinary causes.

**Table 4.** Association Between Birth Defects and Cause-Specific Mortality Before Age 14 Years

Immediate cause of death	No. of deaths (% of children who died)		Hazard ratio (95% CI)^a^
	Children with birth defects	Children without birth defects	
All causes	918 (1.0)	1082 (0.1)	5.11 (4.67-5.59)
Congenital anomalies	200 (0.2)	0	NA
Infection	15 (0.02)	8 (0.001)	14.14 (5.91-33.85)
Cancer	36 (0.04)	34 (0.004)	9.75 (6.07-15.64)
Disease of blood	<5	<5	8.96 (1.23-65.44)
Endocrine, nutritional, metabolic	12 (0.01)	10 (0.001)	11.74 (5.02-27.46)
Nervous system	49 (0.1)	53 (0.01)	8.10 (5.46-12.02)
Circulatory	95 (0.1)	32 (0.003)	26.59 (17.73-39.87)
Respiratory	85 (0.1)	30 (0.003)	23.03 (15.09-35.14)
Digestive	22 (0.02)	5 (0.001)	31.77 (11.87-85.04)
Musculoskeletal and connective tissue	<5	0	NA
Genitourinary	<5	<5	3.34 (0.54-20.55)
Perinatal	330 (0.3)	832 (0.1)	1.95 (1.72-2.22)
Ill-defined conditions	54 (0.1)	42 (0.004)	9.67 (6.40-14.60)
Injury, poisoning, other external causes	14 (0.01)	29 (0.003)	4.10 (2.14-7.84)
Factors associated with health status and contact with health services	<5	<5	4.07 (0.36-46.51)

Abbreviation: NA, not applicable.

^a^ Hazard ratio for any birth defect vs no defect, adjusted for maternal age, parity, multiple birth, preterm birth, sex, socioeconomic deprivation, and period.

Children with birth defects had a high risk of mortality at all ages, especially the postneonatal period (Table 5; eTable 5 in the Supplement). Compared with children with no defect, those with any birth defect had 0.97 times the risk of mortality in the first day of life (95% CI, 0.80-1.19 times), 6.59 times the risk between 1 and 27 days (95% CI, 5.58-7.78 times), 21.22 times the risk between 28 and 364 days (95% CI, 16.98-26.53 times), 13.13 times the risk between 1 and 4 years (95% CI, 10.04-17.15 times), and 11.56 times the risk between 5 and 14 years (95% CI, 7.48-17.88 times). This trend was present for most birth defects, although the risk of mortality was greatest between ages 5 and 14 years for central nervous system defects.

**Table 5.** Association Between Birth Defects and Mortality According to Age at Death

Type of defect^a^	Hazard ratio (95% CI)^b^				
	<1 d	1-27 d	28-364 d	1-4 y	5-14 y
Any	0.97 (0.80-1.19)	6.59 (5.58-7.78)	21.22 (16.98-26.53)	13.13 (10.04-17.15)	11.56 (7.48-17.88)
Heart	0.70 (0.50-0.97)	12.82 (10.61-15.48)	67.66 (52.90-86.54)	38.36 (27.93-52.69)	20.24 (11.08-36.95)
Critical	4.74 (2.68-8.40)	59.37 (44.58-79.08)	289.88 (214.11-392.46)	114.32 (70.91-184.30)	57.28 (20.32-161.47)
Noncritical	0.54 (0.37-0.78)	11.81 (9.75-14.30)	66.45 (51.86-85.15)	39.04 (28.42-53.64)	20.62 (11.28-37.66)
Central nervous system	1.55 (1.01-2.38)	10.75 (8.02-14.42)	62.62 (44.40-88.32)	98.76 (68.44-142.50)	182.07 (110.11-301.06)
Orofacial cleft	1.06 (0.25-4.55)	13.18 (7.20-24.11)	60.26 (36.00-100.86)	31.11 (13.61-71.09)	12.64 (1.73-92.34)
Eye, ear, nose	0.36 (0.15-0.86)	2.89 (1.77-4.72)	12.79 (8.37-19.55)	10.99 (6.69-18.03)	2.43 (0.59-10.11)
Respiratory	39.43 (33.35-46.62)	9.36 (6.50-13.49)	34.38 (22.66-52.18)	48.20 (29.81-77.91)	32.97 (13.44-80.86)
Digestive	0.44 (0.18-1.11)	9.06 (6.53-12.58)	46.73 (33.69-64.81)	31.05 (19.47-49.52)	25.00 (11.41-54.79)
Abdominal wall	3.18 (1.79-5.64)	21.30 (14.47-31.36)	39.96 (22.93-69.65)	32.36 (12.91-81.17)	13.06 (1.73-98.74)
Urinary	1.11 (0.70-1.78)	4.90 (3.43-6.99)	22.08 (15.58-31.30)	17.88 (11.34-28.19)	8.61 (3.35-22.10)
Genital	0.19 (0.05-0.73)	3.14 (1.85-5.32)	7.51 (4.07-13.85)	10.05 (4.97-20.33)	5.70 (1.73-18.79)
Musculoskeletal	1.05 (0.69-1.60)	4.23 (3.04-5.87)	14.38 (10.22-20.24)	12.51 (8.40-18.62)	7.32 (3.40-15.79)
Chromosomal	6.09 (4.19-8.87)	19.29 (13.47-27.61)	91.79 (62.63-134.54)	101.13 (63.88-160.10)	86.80 (39.03-193.04)
None	1 [Reference]	1 [Reference]	1 [Reference]	1 [Reference]	1 [Reference]

^a^ Categories are not mutually exclusive.

^b^ Hazard ratio for birth defect vs no defect, adjusted for maternal age, parity, multiple birth, preterm birth, sex, socioeconomic deprivation, and period.

In sensitivity analyses, excluding preterm births strengthened the associations. Relative to that of children with no defect, risk of death for boys with birth defects was 14.66 times greater (95% CI, 11.88-18.09); and for girls, 18.44 times greater (95% CI, 14.71-23.11).

## Discussion

In this study of 1 037 688 children in Canada, birth defects were associated with more than 4.7 times the risk of death before age 14 years. Nearly half of children who died had a birth defect. Central nervous system defects were associated with more than 10 times the risk of death; heart defects, with more than 8 times the risk; and chromosomal anomalies, with more than 17 times the risk. Children with birth defects had an elevated risk of death from circulatory, respiratory, and digestive causes. Risk of death between 28 and 364 days of life was considerably elevated, although there was an elevated risk at other points as well. The findings demonstrate that in children with birth defects, the cause of death is frequently a reason other than the anomaly itself. The contribution of birth defects may be underestimated in mortality data.

In current statistics, deaths owing to birth defects are frequently captured with vital statistics or other cross-sectional information.^2,7,8,9^ Birth defects are ascertained only if the anomaly is the obvious cause of death.^14^ A study of 83 183 children with death certificates in Michigan found that underlying birth defects accounted for only 18% of deaths.^6^ The proportion increased to 34% when the authors used longitudinal data, suggesting that death certificates may underestimate the contribution of defects to mortality.^6^ An analysis of death certificates revealed that birth defects were the underlying cause of death in less than 83% of children with known anomalies.^14^ In our longitudinal cohort, nearly half of children who died had birth defects, but only 22% had an anomaly recorded as the cause of death.

Birth defects were less often recorded as the cause of death in our data, contrasting with birth cohorts from the 1970s and 1980s, in which anomalies accounted for two-thirds of deaths.^5^ The majority of children with birth defects in our cohort ultimately died from causes potentially related to the birth defect, including complications arising in the perinatal period. Up to 38% of deaths among children with birth defects were due to perinatal conditions. Improvements in early neonatal life support may have been associated with an increased number of deaths from these causes over time.^15,16,17,18^

Risk of death was high for circulatory, respiratory, and digestive causes. Compared with no defect, birth defects were associated with more than 23 times the risk of mortality from these causes. In Texas, a study of 8000 infants with birth defects found that deaths owing to circulatory, respiratory, and digestive causes were frequent before 1 year of age.^19^ In another study of children with birth defects, circulatory disorders ranked as an important cause of death before age 10 years.^6^ Circulatory disorders are common in children with heart defects, diaphragmatic hernia, and lung hypoplasia.^19^ Infections such as respiratory syncytial virus can be common in children with spina bifida, lung malformations, cleft palate, and biliary atresia.^20^ Prematurity can also exacerbate risks of mortality because of circulatory, respiratory, and digestive complications,^17,19,21^ although birth defects were associated with these causes even when we excluded preterm births.

Defects such as hypoplastic left heart syndrome, trisomy 13 and 18, and anencephaly are known for causing high mortality.^3,4,10,13^ These defects were associated with more than 160 times the risk of death in a study of 262 352 children from New York.^13^ They were also fairly lethal in our cohort, although the magnitude of association was lower. However, our data covered a later period, and improvements in clinical management may have been associated with increased survival of children with hypoplastic left heart syndrome and chromosomal anomalies.^18,22,23^ Most children with anencephaly die by 1 month of age, although survival past 2 years has been documented.^4,24^

Children with birth defects had a markedly greater risk of death in the postneonatal period, or between 28 and 364 days of life. Birth defects were associated with 6.6 times the risk of death between 1 and 27 days, but 21.2 times the risk between 28 and 364 days. This pattern suggests that deaths of children with birth defects who would otherwise die without life-sustaining interventions may be displaced to the postneonatal period.^25^ Displaced deaths may ultimately be unavoidable owing to a high prevalence of morbidities.^26^ Deaths are less likely to be displaced in children without defects, in whom the majority of deaths are concentrated in the earliest days of life.

Today, most children with heart defects are expected to reach adulthood. Survival may depend on severity of the defect, age at surgery, and other prognostic factors.^27^ In our data, children with heart, respiratory, and chromosomal anomalies continued to be at risk of death in infancy and childhood, although mortality rates decreased after the postneonatal period. Children with heart defects had nearly 40 times the risk between ages 1 and 4 years and 20 times the risk between 5 and 14 years compared with those with no defect. Children with respiratory or chromosomal anomalies had 33 to 87 times the risk of death between ages 5 and 14 years. Many children with lung malformations are initially asymptomatic before presenting with infections later in infancy.^28^ Prophylactic lobectomy is possible, but the association with survival remains uncertain.^28^ Careful monitoring may be needed, especially for cardiorespiratory and chromosomal anomalies, because in our cohort these children had a high prevalence of mortality from circulatory and respiratory causes.

For central nervous system defects, mortality rates did not improve substantially with age. Risk of death was higher after age 1 year and peaked between 5 and 14 years. A longitudinal study of 117 infants with surgically treated spina bifida found that mortality rates remained high into adulthood.^29^ Complications related to surgery or medical devices, motor or sensory deficiency, or bladder, bowel, and cognitive impairment are common with spina bifida.^29,30^ Sepsis, pneumonia, and respiratory failure can account for more than one-third of hospitalizations that lead to death.^30^ In our data, respiratory disorders were a common cause of death in children with central nervous system defects.

### Limitations

This cohort study had a large sample size and included birth defects that were detected later in childhood, but there are limitations. We used administrative data in which coding errors may have misclassified exposures or outcomes and attenuated results toward the null. We lacked data on birth defects diagnosed in ambulatory clinics, and potential confounders such as race and ethnicity. We had limited statistical power to examine associations for rare birth defects and uncommon causes of death. We used data that provided the immediate cause of death, information that is important for mortality prevention but may not reflect the sequence of events that lead to death. Finally, the results may not be generalizable to settings without a high standard of health care because the cohort comprised children with publicly funded health insurance in Canada.

## Conclusions

This longitudinal cohort study of 1 037 688 Canadian children suggests that birth defects are associated with a high risk of mortality in the postneonatal period, especially mortality due to circulatory, respiratory, and digestive causes. Among children with birth defects, perinatal conditions were the most common cause of death, whereas birth defects were recorded for a smaller fraction of deaths. The findings suggest that birth defects are substantially associated with child mortality despite medical advances. Better management of cardiorespiratory and digestive causes of mortality may be needed to improve long-term survival of children with birth defects. More accurate mortality statistics are required to inform guidelines for prevention of mortality in children with birth defects.

## Notes

Supplement. **eTable 1.** STROBE Checklist **eTable 2.** Diagnostic and Procedural Codes for Birth Defects **eTable 3.** Descriptive Characteristics of Children With and Without Birth Defects **eTable 4.** Unadjusted Association Between Birth Defects and Mortality Before Age 14 Years **eTable 5.** Mortality Rate According to Age at Death

## References

[1] Glinianaia SV, Morris JK, Best KE, . Long-term survival of children born with congenital anomalies: a systematic review and meta-analysis of population-based studies. PLoS Med. 2020;17(9):e1003356. doi:10.1371/journal.pmed.1003356 32986711 PMC7521740

[2] Almli LM, Ely DM, Ailes EC, . Infant mortality attributable to birth defects—United States, 2003-2017. MMWR Morb Mortal Wkly Rep. 2020;69(2):25-29. doi:10.15585/mmwr.mm6902a1 31945037 PMC6973351

[3] Best KE, Rankin J. Long-term survival of individuals born with congenital heart disease: a systematic review and meta-analysis. J Am Heart Assoc. 2016;5(6):e002846. doi:10.1161/JAHA.115.002846 27312802 PMC4937249

[4] Blencowe H, Kancherla V, Moorthie S, Darlison MW, Modell B. Estimates of global and regional prevalence of neural tube defects for 2015: a systematic analysis. Ann N Y Acad Sci. 2018;1414(1):31-46. doi:10.1111/nyas.13548 29363759

[5] Agha MM, Williams JI, Marrett L, To T, Dodds L. Determinants of survival in children with congenital abnormalities: a long-term population-based cohort study. Birth Defects Res A Clin Mol Teratol. 2006;76(1):46-54. doi:10.1002/bdra.20218 16397887

[6] Copeland GE, Kirby RS. Using birth defects registry data to evaluate infant and childhood mortality associated with birth defects: an alternative to traditional mortality assessment using underlying cause of death statistics. Birth Defects Res A Clin Mol Teratol. 2007;79(11):792-797. doi:10.1002/bdra.20391 17990340

[7] Mathews TJ, Driscoll AK. Trends in infant mortality in the United States, 2005-2014. NCHS Data Brief. 2017;(279):1-8. 28437240

[8] Petrini J, Damus K, Russell R, Poschman K, Davidoff MJ, Mattison D. Contribution of birth defects to infant mortality in the United States. Teratology. 2002;66(suppl 1):S3-S6. doi:10.1002/tera.90002 12239736

[9] Zhu Y, Zhu X, Deng M, Wei H, Zhang M. Causes of death in hospitalized children younger than 12 years of age in a Chinese hospital: a 10 year study. BMC Pediatr. 2018;18(1):8. doi:10.1186/s12887-017-0981-y 29347924 PMC5773040

[10] Meyer RE, Liu G, Gilboa SM, ; National Birth Defects Prevention Network. Survival of children with trisomy 13 and trisomy 18: a multi-state population-based study. Am J Med Genet A. 2016;170A(4):825-837. doi:10.1002/ajmg.a.37495 26663415 PMC4898882

[11] Ministry of Health and Social Services. Med-Echo System Normative Framework—Maintenance and Use of Data for the Study of Hospital Clientele. Government of Quebec; 2021.

[12] Boissière-O’Neill T, Schnitzer ME, Lewin A, Bilodeau-Bertrand M, Ayoub A, Auger N. Original article: is the protective association between hyperemesis gravidarum and birth defects biased by pregnancy termination? Ann Epidemiol. 2021;59:10-15. doi:10.1016/j.annepidem.2021.03.007 33798708

[13] Wang Y, Hu J, Druschel CM. A retrospective cohort study of mortality among children with birth defects in New York State, 1983-2006. Birth Defects Res A Clin Mol Teratol. 2010;88(12):1023-1031. doi:10.1002/bdra.20711 20683907

[14] Hoyert DL. Mortality associated with birth defects: influence of successive disease classification revisions. Birth Defects Res A Clin Mol Teratol. 2003;67(9):651-655. doi:10.1002/bdra.10117 14703789

[15] Bakker MK, Kancherla V, Canfield MA, . Analysis of mortality among neonates and children with spina bifida: an international registry-based study, 2001-2012. Paediatr Perinat Epidemiol. 2019;33(6):436-448. doi:10.1111/ppe.12589 31637749 PMC6899817

[16] Liu S, Joseph KS, Kramer MS, ; Fetal and Infant Health Study Group of the Canadian Perinatal Surveillance System. Relationship of prenatal diagnosis and pregnancy termination to overall infant mortality in Canada. JAMA. 2002;287(12):1561-1567. doi:10.1001/jama.287.12.1561 11911759

[17] Honein MA, Kirby RS, Meyer RE, ; National Birth Defects Prevention Network. The association between major birth defects and preterm birth. Matern Child Health J. 2009;13(2):164-175. doi:10.1007/s10995-008-0348-y 18484173 PMC11669608

[18] Sadowski SL. Congenital cardiac disease in the newborn infant: past, present, and future. Crit Care Nurs Clin North Am. 2009;21(1):37-48, vi. doi:10.1016/j.ccell.2008.10.001 19237042

[19] Benjamin RH, Salemi JL, Canfield MA, . Causes of neonatal and postneonatal death among infants with birth defects in Texas. Birth Defects Res. 2021;113(9):665-675. doi:10.1002/bdr2.1879 33586914 PMC8920027

[20] Zachariah P, Ruttenber M, Simões EAF. Hospitalizations due to respiratory syncytial virus in children with congenital malformations. Pediatr Infect Dis J. 2011;30(5):442-445. doi:10.1097/INF.0b013e318201813b 21127456

[21] Dalla Vecchia LK, Grosfeld JL, West KW, Rescorla FJ, Scherer LR, Engum SA. Intestinal atresia and stenosis: a 25-year experience with 277 cases. Arch Surg. 1998;133(5):490-496. doi:10.1001/archsurg.133.5.490 9605910

[22] Peterson JK, Kochilas LK, Catton KG, Moller JH, Setty SP. Long-term outcomes of children with trisomy 13 and 18 after congenital heart disease interventions. Ann Thorac Surg. 2017;103(6):1941-1949. doi:10.1016/j.athoracsur.2017.02.068 28456396 PMC5526352

[23] Knowles RL, Bull C, Wren C, Dezateux C. Mortality with congenital heart defects in England and Wales, 1959-2009: exploring technological change through period and birth cohort analysis. Arch Dis Child. 2012;97(10):861-865. doi:10.1136/archdischild-2012-301662 22753769

[24] Byrne P; Canadian Paediatric Society (CPS), Bioethics Committee. Use of anencephalic newborns as organ donors. Paediatr Child Health. 2005;10(6):335-337. doi:10.1093/pch/10.6.335 19675842 PMC2722973

[25] Auger N, Bilodeau-Bertrand M, Nuyt AM. Dangers of death on the first day of life by the minute. J Perinatol. 2015;35(11):958-964. doi:10.1038/jp.2015.107 26334397

[26] Nguyen JE, Salemi JL, Tanner JP, . Survival and healthcare utilization of infants diagnosed with lethal congenital malformations. J Perinatol. 2018;38(12):1674-1684. doi:10.1038/s41372-018-0227-3 30237475

[27] Spector LG, Menk JS, Knight JH, . Trends in long-term mortality after congenital heart surgery. J Am Coll Cardiol. 2018;71(21):2434-2446. doi:10.1016/j.jacc.2018.03.491 29793633 PMC5978758

[28] Annunziata F, Bush A, Borgia F, . Congenital lung malformations: unresolved issues and unanswered questions. Front Pediatr. 2019;7:239. doi:10.3389/fped.2019.00239 31249823 PMC6584787

[29] Oakeshott P, Hunt GM, Poulton A, Reid F. Expectation of life and unexpected death in open spina bifida: a 40-year complete, non-selective, longitudinal cohort study. Dev Med Child Neurol. 2010;52(8):749-753. doi:10.1111/j.1469-8749.2009.03543.x 20015251

[30] Dicianno BE, Wilson R. Hospitalizations of adults with spina bifida and congenital spinal cord anomalies. Arch Phys Med Rehabil. 2010;91(4):529-535. doi:10.1016/j.apmr.2009.11.023 20382283 PMC8474055

