# Supplementary material for: Association of Birth Defects With Child Mortality Before Age 14 Years
Source: JAMA Netw Open. 2022 Apr 11;5(4):e226739. doi: 10.1001/jamanetworkopen.2022.6739 (PMC9002336; doi:10.1001/jamanetworkopen.2022.6739)
Supplement: Supplement. — eTable 1. STROBE Checklist eTable 2. Diagnostic and Procedural Codes for Birth Defects eTable 3. Descriptive Characteristics of Children With and Without Birth Defects eTable 4. Unadjusted Association Between Birth Defects and Mortality Before Age 14 Years eTable 5. Mortality Rate According to Age at Death [file jamanetwopen-e226739-s001.pdf]

Supplemental Online Content

Sattolo ML, Arbour L, Bilodeau-Bertrand M, Lee GE, Nelson C, Auger N. Association of birth defects with child mortality before age 14 years. *JAMA Netw Open*. 2022;5(4):e226739. doi:10.1001/jamanetworkopen.2022.6739

eTable 1. STROBE Checklist
eTable 2. Diagnostic and Procedural Codes for Birth Defects
eTable 3. Descriptive Characteristics of Children With and Without Birth Defects
eTable 4. Unadjusted Association Between Birth Defects and Mortality Before Age 14 Years
eTable 5. Mortality Rate According to Age at Death

This supplemental material has been provided by the authors to give readers additional information about their work.

© 2022 Sattolo ML et al. *JAMA Network Open.*

eTable 1. STROBE Checklist

	Item No	Recommendation	Page No
**Title and abstract**	1	(*a*) Indicate the study's design with a commonly used term in the title or the abstract	3
		(*b*) Provide in the abstract an informative and balanced summary of what was done and what was found	3
**Introduction**			
Background/rationale	2	Explain the scientific background and rationale for the investigation being reported	5
Objectives	3	State specific objectives, including any prespecified hypotheses	5
**Methods**			
Study design	4	Present key elements of study design early in the paper	6
Setting	5	Describe the setting, locations, and relevant dates, including periods of recruitment, exposure, follow-up, and data collection	6-8
Participants	6	(*a*) Give the eligibility criteria, and the sources and methods of selection of participants. Describe methods of follow-up	6-8
		(*b*) For matched studies, give matching criteria and number of exposed and unexposed	N/A
Variables	7	Clearly define all outcomes, exposures, predictors, potential confounders, and effect modifiers. Give diagnostic criteria, if applicable	6-8
Data sources/ measurement	8	For each variable of interest, give sources of data and details of methods of assessment (measurement). Describe comparability of assessment methods if there is more than one group	6-8
Bias	9	Describe any efforts to address potential sources of bias	6,8,9
Study size	10	Explain how the study size was arrived at	6
Quantitative variables	11	Explain how quantitative variables were handled in the analyses. If applicable, describe which groupings were chosen and why	8-9
Statistical methods	12	(*a*) Describe all statistical methods, including those used to control for confounding	8-9
		(*b*) Describe any methods used to examine subgroups and interactions	8-9
		(*c*) Explain how missing data were addressed	8
		(*d*) If applicable, explain how loss to follow-up was addressed	8
		(*e*) Describe any sensitivity analyses	8-9

© 2022 Sattolo et al. *JAMA Network Open.*

**Results**			
Participants	13	(a) Report numbers of individuals at each stage of study—eg numbers potentially eligible, examined for eligibility, confirmed eligible, included in the study, completing follow-up, and analysed	9
		(b) Give reasons for non-participation at each stage	N/A
		(c) Consider use of a flow diagram	N/A
Descriptive data	14	(a) Give characteristics of study participants (eg demographic, clinical, social) and information on exposures and potential confounders	9
		(b) Indicate number of participants with missing data for each variable of interest	8
		(c) Summarise follow-up time (eg, average and total amount)	9
Outcome data	15	Report numbers of outcome events or summary measures over time	9-10
Main results	16	(*a*) Give unadjusted estimates and, if applicable, confounder-adjusted estimates and their precision (eg, 95% confidence interval). Make clear which confounders were adjusted for and why they were included	9-11
		(*b*) Report category boundaries when continuous variables were categorized	11
		(*c*) If relevant, consider translating estimates of relative risk into absolute risk for a meaningful time period	N/A
Other analyses	17	Report other analyses done—eg analyses of subgroups and interactions, and sensitivity analyses	11
**Discussion**			
Key results	18	Summarise key results with reference to study objectives	11-12
Limitations	19	Discuss limitations of the study, taking into account sources of potential bias or imprecision. Discuss both direction and magnitude of any potential bias	15
Interpretation	20	Give a cautious overall interpretation of results considering objectives, limitations, multiplicity of analyses, results from similar studies, and other relevant evidence	11-15
Generalisability	21	Discuss the generalisability (external validity) of the study results	15
**Other information**			
Funding	22	Give the source of funding and the role of the funders for the present study and, if applicable, for the original study on which the present article is based	17

© 2022 Sattolo et al. *JAMA Network Open.*

eTable 2. Diagnostic and Procedural Codes for Birth Defects

	International Classification of Diseases 10th Revision / Canadian Classification of Health Interventions
**Critical heart defect**	
Transposition of great vessels	Q20.1-Q20.3, Q20.50
Tetralogy of Fallot	Q21.3 / 1.LD.84, 1.HP.87
Hypoplastic left heart	Q23.4
Coarctation of aorta	Q25.1
Other critical	Q20.0, Q20.4, Q21.4, Q22.0, Q22.4, Q22.5, Q26.2 / 1.LA.84
**Noncritical heart defect**	
Ventricular septum	Q21.0, Q21.8 / 1.HR.80
Atrial septum	Q21.1 / 1.HN.80
Other noncritical	Q20.58, Q20.6-Q20.9, Q21.2, Q21.9, Q22.1-Q22.3, Q22.6, Q22.8-Q23.3, Q23.8-Q25.0, Q25.2-Q26.1, Q26.3-Q28, Q89.3 / 1.LC.84
**Central nervous system**	
Neural tube defects	Q00, Q01, Q05, Q07.0 / 1.AW.72, 1.AX.73
Microcephaly	Q02
Hydrocephalus	Q03 / 1.AC.52-1.AC.54
Other central nervous system	Q04, Q06, Q07.8-Q07.9
**Orofacial cleft**	Q35-Q37 / 1.FB.86, 1.YE.80
**Eye, ear, nose**	
Eye	Q10-Q15, H26 / 1.CL.59, 1.CL.89
Ear	Q16-Q17
Nose	Q30
**Respiratory**	
Lung malformation	Q33
Other respiratory	Q31, Q32, Q34
**Digestive**	
Biliary or intestinal atresia	Q39.0, Q39.1, Q41, Q42, Q44.2, Q44.3
Other digestive	K31.1, Q38.0, Q38.2-Q38.8, Q39.2-Q40, Q43-Q44.1, Q44.4-Q45 / 1.NE.72
**Abdominal wall**	
Diaphragmatic hernia	Q79.0
Omphalocele	Q79.2
Gastroschisis	Q79.3 / 1.SY.84
Other abdominal wall defect	Q79.1, Q79.5
**Urinary**	
Renal agenesis	Q60
Other urinary defect	Q61-Q63, Q64.1-Q64.9
**Genital**	
Female genital defect	Q50.0, Q50.3, Q50.4, Q50.6, Q51-Q52.2, Q52.4, Q52.6, Q52.8, Q52.9
Male genital defect	Q54, Q55, Q64.0
Indeterminate sex	Q56

© 2022 Sattolo et al. *JAMA Network Open.*

**Musculoskeletal**	
Congenital hip dislocation	Q65
Clubfoot	Q66 / 1.WE.72
Polydactyly, syndactyly	Q69, Q70 / 1.UF.84, 1.UI.71, 1.UJ.71, 1.WL.71
Limbs and digits	Q71.0-Q71.5, Q71.8-Q73
Other musculoskeletal	Q68.1-Q68.8, Q71.6, Q74-Q75.2, Q75.4-Q75.9, Q76.1-Q76.3, Q76.8-Q78, Q79.4, Q79.6-Q79.9
**Chromosomal**	
Down syndrome	Q90
Trisomy 13 and 18	Q91
Other chromosomal	Q92-Q99
**Other**	Q80-Q82.4, Q82.9-Q83.2, Q83.8-Q84.4, Q84.8-Q87, Q89.0-Q89.2, Q89.4-Q89.8

© 2022 Sattolo et al. *JAMA Network Open.*

eTable 3. Descriptive Characteristics of Children With and Without Birth Defects

	**No. infants (%)**	
	**Birth defect**	**No defect**
**Maternal age, years**		
<25	15,145 (15.8)	143,558 (15.2)
25-34	63,849 (66.8)	627,596 (66.6)
≥35	16,572 (17.3)	170,968 (18.1)
**Parity**		
0	48,927 (51.2)	460,301 (48.9)
1	31,938 (33.4)	328,066 (34.8)
≥2	14,701 (15.4)	153,755 (16.3)
**Multiple birth**		
Yes	3,004 (3.1)	17,493 (1.9)
No	92,562 (96.9)	924,629 (98.1)
**Preterm birth, weeks**		
<37	12,560 (13.1)	57,238 (6.1)
≥37	83,006 (86.9)	884,884 (93.9)
**Sex**		
Male	53,954 (56.5)	478,588 (50.8)
Female	41,612 (43.5)	463,534 (49.2)
**Socioeconomic deprivation**		
Yes	18,928 (19.8)	189,283 (20.1)
No	72,909 (76.3)	714,934 (75.9)
**Time period**		
2006-2009	29,486 (30.9)	277,691 (29.5)
2010-2014	37,352 (39.1)	370,107 (39.3)
2015-2019	28,728 (30.1)	294,324 (31.2)
**Total**	95,566 (100.0)	942,122 (100.0)

© 2022 Sattolo et al. *JAMA Network Open.*

eTable 4. Unadjusted Association Between Birth Defects and Mortality Before Age 14 Years

**Type of birth defect[a]**	**Unadjusted hazard ratio (95% CI)**	
	**Boys**	**Girls**
Any defect	7.46 (6.62-8.40)	9.56 (8.39-10.88)
Heart	24.11 (21.00-27.69)	28.63 (24.75-33.10)
Central nervous	36.40 (29.77-44.52)	41.62 (33.60-51.55)
Orofacial cleft	16.37 (10.59-25.29)	17.48 (10.63-28.76)
Eye, ear, nose	3.57 (2.58-4.94)	3.54 (2.43-5.14)
Respiratory	19.87 (15.32-25.76)	24.85 (18.48-33.42)
Digestive	16.70 (13.24-21.07)	21.15 (16.21-27.59)
Abdominal wall[b]	41.01 (29.29-57.41)	39.18 (26.78-57.32)
Urinary	6.92 (5.46-8.78)	10.63 (7.99-14.14)
Genital	3.60 (2.57-5.04)	3.70 (1.38-9.89)
Musculoskeletal	5.42 (4.31-6.82)	4.70 (3.66-6.05)
Chromosomal	48.51 (37.98-61.95)	56.83 (43.95-73.47)
Other	21.01 (16.62-26.58)	26.83 (21.08-34.16)
Multiple defects	29.60 (25.39-34.52)	41.92 (35.43-49.61)
Isolated defect	4.59 (3.98-5.31)	5.93 (5.08-6.93)
No defect	Reference	Reference

[a]Categories are not mutually exclusive.
[b]Gastroschisis, omphalocele, diaphragmatic hernia, other.

© 2022 Sattolo et al. *JAMA Network Open.*

eTable 5. Mortality Rate According to Age at Death

	No. deaths					Mortality rate per 1,000 children				
**Type of defect[a]**	**<1 day**	**1-27 days**	**28-364 days**	**1-4 years**	**5-14 years**	**<1 day**	**1-27 days**	**28-364 days**	**1-4 years**	**5-14 years**
Any defect	117	312	307	133	49	1.2	3.3	3.2	1.4	0.7
Heart defect	38	227	232	78	19	1.9	11.4	11.7	4.0	1.5
Critical	12	58	73	21	<5	6.3	30.8	39.9	12.0	3.4
Noncritical	29	212	224	78	19	1.5	10.8	11.5	4.1	1.5
Central nervous	15	56	62	49	32	3.1	11.6	12.9	10.4	10.0
Orofacial cleft	<5	11	17	6	<5	1.0	5.8	9.0	3.2	0.8
Eye, ear, nose	<5	17	27	19	<5	0.2	1.0	1.6	1.2	0.2
Respiratory	19	33	31	22	6	4.3	7.4	7.0	5.0	2.2
Digestive	8	42	61	23	8	1.2	6.3	9.2	3.5	1.7
Abdominal wall	12	30	16	5	<5	8.5	21.5	11.7	3.7	1.1
Urinary	17	35	48	24	5	1.2	2.6	3.5	1.8	0.6
Genital	<5	15	12	9	<5	0.1	1.6	1.3	1.0	0.5
Musculoskeletal	22	41	48	33	8	0.8	1.6	1.8	1.3	0.4
Chromosomal	29	35	41	25	8	12.4	15.2	18.0	11.2	5.3
No defect	573	271	108	94	36	0.6	0.3	0.1	0.1	0.1

[a]Categories are not mutually exclusive.

© 2022 Sattolo et al. *JAMA Network Open.*
